# Analysis of novel endosome-to-Golgi retrieval genes reveals a role for PLD3 in regulating endosomal protein sorting and amyloid precursor protein processing

**DOI:** 10.1007/s00018-018-2752-9

**Published:** 2018-01-24

**Authors:** Aamir S. Mukadam, Sophia Y. Breusegem, Matthew N. J. Seaman

**Affiliations:** Cambridge Institute for Medical Research, Cambridge Biomedical Campus, University of Cambridge, Wellcome Trust/MRC Building, Hills Road, Cambridge, CB2 0XY UK

**Keywords:** Phospholipase D, Endosome, Amyloid precursor protein, SorL1, Alzheimer disease

## Abstract

The processing of amyloid precursor protein (APP) to the neurotoxic pro-aggregatory Aβ peptide is controlled by the mechanisms that govern the trafficking and localisation of APP. We hypothesised that genes involved in endosomal protein sorting could play an important role in regulating APP processing and, therefore, analysed ~ 40 novel endosome-to-Golgi retrieval genes previously identified in a genome-wide siRNA screen. We report that phospholipase D3 (PLD3), a type II membrane protein, functions in endosomal protein sorting and plays an important role in regulating APP processing. PLD3 co-localises with APP in endosomes and loss of PLD3 function results in reduced endosomal tubules, impaired trafficking of several membrane proteins and reduced association of sortilin-like 1 with APP.

**Electronic supplementary material:**

The online version of this article (10.1007/s00018-018-2752-9) contains supplementary material, which is available to authorized users.

## Introduction

The processing of amyloid precursor protein (APP) to form the neurotoxic pro-aggregatory Aβ peptide is believed to be a key initiating event in the pathogenesis of Alzheimer’s disease [1]. The trafficking and localisation of APP within the post-Golgi endocytic system plays an important role in regulating the exposure of APP to the secretases that mediate its cleavage to form Aβ peptides [2, 3]. Most evidence now supports a model whereby the cleavage of APP to generate Aβ occurs in an endocytic compartment where β-secretase (BACE) is predominately localised [4]. Thus, mechanisms that direct APP away from endosomes towards either the Golgi complex or the cell surface are considered to be protective of APP processing to Aβ [5, 6].

The retromer complex is a key mediator of endosomal protein sorting and has been shown to operate in both endosome-to-Golgi and endosome-to-cell surface traffic, regulating the localisation of membrane proteins such as the cation-independent mannose 6-phosphate receptor (CIMPR), sortilin and Glut-1 [7]. The retromer complex comprises a stable trimeric protein complex containing Vps35, Vps26 and Vps29 that together select membrane proteins (‘cargo’) for packaging into tubular carriers that form through the action of the other functional unit of retromer, the sorting nexin (Snx) dimer. For endosome-to-Golgi traffic, the Snx dimer contains one copy of either Snx1 or Snx2 paired with either Snx5 or Snx6 [8, 9].

Another notable cargo protein for retromer is the membrane protein SorL1 (also known as SorLA) that traffics from endosomes to the Golgi in a retromer-dependent manner. SorL1 can directly associate with APP and can bind to the retromer cargo-selective trimer through an interaction with Vps26. SorL1 can thereby direct APP into the retromer-mediated endosome-to-Golgi retrieval pathway thus protecting APP from cleavage by BACE [10–12]. Mutations in SorL1 can cause late-onset AD (LOAD) [13, 14] and variants of genes that regulate recruitment of the retromer complex to endosomes have been shown to predispose to LOAD [15]. Furthermore, a point mutation in the retromer protein, Vps35, that results in the protein becoming unstable, may be causal in early-onset AD [16]. Also, it has been shown that expression levels of retromer proteins are reduced in the brains of AD patients and that loss of retromer function results in increased processing of APP to Aβ [17, 18].

Due to its key function in endosomal protein sorting and prominent role in regulating APP trafficking, there has been considerable interest in retromer as a potential engine of pathogenesis for AD [19, 20], but retromer does not operate in isolation in endosomal protein sorting. We have recently reported the results of a genome-wide siRNA screen for novel endosome-to-Golgi retrieval genes that may function alongside retromer [21]. Among the genes identified as new players in the endosome-to-Golgi pathway were a surprising number of multi-pass membrane-spanning proteins including SFT2D2 and ZDHHC5. These two proteins are required for the endosome-to-Golgi retrieval of the CIMPR and both SFT2D2 and ZDHHC5 are localised to endosomes positive for retromer proteins. We hypothesised that any of the genes identified as novel endosome-to-Golgi retrieval genes may encode proteins that could function in endosomal protein sorting and may, therefore, regulate APP localisation and processing. We have undertaken an analysis of the endosome-to-Golgi retrieval genes and identified those genes that, when silenced, result in increased processing of APP to Aβ. We report that among the hits, the PLD3 gene exerts a pronounced effect on Aβ secretion. Furthermore, we show that PLD3 localises to retromer-positive and APP-positive endosomes and regulates the localisation of SorL1 and its association with APP.

## Materials and methods

### Cloning

The full-length PLD3 (WT) open reading frame (ORF) of 490 amino acids was amplified by polymerase chain reaction (PCR) using primers to introduce Bam HI and Sal I restriction enzyme sites to the 5′ and 3′ ends, respectively. All PCR products were first subcloned using the PCR blunt vector (Invitrogen) and sequenced. The digested ORFs were then subcloned into the Bgl II and Sal I sites of the pEGFP-N3 vector (CLONTECH) for expression as a GFP-fusion protein in mammalian cells.

### Western blotting

Cells were harvested with a sterile cell scraper and lysed in lysis buffer (20 mM HEPES–KOH, pH 7.2, 50 mM potassium acetate, 2 mM EDTA, 200 mM sorbitol, 1% Triton X-100, 0.1% SDS) containing Halt™ Protease Inhibitor Cocktail (Thermo Scientific). Cell debris was removed by centrifugation at 20,000×*g*, 4 °C for 10 min. Supernatants were transferred to fresh microfuge tubes, and to an aliquot of the lysate appropriate volumes of 4× NuPAGE LDS sample buffer (Life Technologies) containing 50 mM DTT was added and heated to either 95 °C for 5 min or 70 °C for 10 min. Samples were resolved using NuPAGE Bis–Tris Novex 4–12% gels (Life Technologies) and electroblotted to a 0.2-μm PVDF membrane using the Transblot Turbo Transfer System (Bio-Rad). Membranes were blocked with 5% milk TBS–Tween 20 before incubation with primary antibodies overnight at 4 °C. Membranes were then probed with appropriate secondary antibodies conjugated with HRP for 1 h. Membranes were washed repeatedly in TBS–0.1% Tween-20 after both primary and secondary antibody incubation. Blots were incubated with Pierce Super Signal or Millipore Immobilon enhanced chemiluminescence reagents for 5 min and exposed to X-ray film and developed or visualised using a ChemiDoc system (Bio-Rad).

### Aβ detection

For Aβ detection, appropriate volumes of 4× LDS sample buffer with 50 mM DTT were added to conditioned cell culture media that had been spun for 2 min at 2000 rpm and then heated at 95 °C for 5 min. Samples were then processed as described above. For detecting CTFβ and Aβ, the PVDF membrane was boiled post-transfer in pre-warmed PBS for 5 min prior to blocking. Membranes were processed as described above.

### Antibodies

Anti-M6PR (cation independent) antibody [2G11] (ab2733; Abcam).

Anti-APP (A8717; Sigma).

Anti-APP 6e10 to detect Aβ, sAPPα (SIG-39320; Covance).

Anti-GAPDH (Sigma).

Anti-tubulin (Sigma).

Anti-cyclophilin B (ACB0825; Atgen).

Anti-sAPPβ Swedish (6A-1; IBL).

Anti-VPS35 (SC-374372; Santa Cruz).

Anti-LAMP-1 (SC-18821; Santa Cruz).

Anti-PLD3 (HPA012800; Sigma).

Anti-transferrin receptor (13-6800; Invitrogen).

Anti-SORL1 (612633; BD Bioscience).

Anti-GFP (Seaman lab).

Anti-LBPA (MABT837; Sigma).

Anti-EEA1 (610456; BD Biosciences).

Anti-GM130 (610822; BD Biosciences).

Anti-TGN46 (Seaman lab).

Anti-MICALL1 (H00085377-B01P, Novus).

Anti-SNAP29 (gift from Andrew Peden, University of Sheffield, UK).

Anti-PACSIN2 (ab37615, Abcam).

### Sucrose gradients

All sucrose solutions were made wt/wt (%) with ultra-pure sucrose in 20 mM HEPES–KOH, pH 7.2, 50 mM potassium acetate, 1 mM EDTA, 1 mM DTT. The gradients were poured in a series of steps: 1 mL 60%, 1.0 mL 37%, 1.5 mL 34%, 2.0 mL 32%, 2.0 mL 29%, 1.0 mL 27%, 1.5 mL 22%, and 0.5 mL 10%.

Cells were washed with ice-cold PBS and then resuspended in an ice-cold lysis buffer (20 mM HEPES–KOH, pH 6.8, 50 mM potassium acetate, 1 mM EDTA, 200 mM sorbitol, 1 mM DTT). The resulting suspension was Dounce homogenized in an ice-cold tissue homogenizer 15–20 times and then subjected to centrifugation at 500×*g* (5 min) to remove unbroken cells. Samples were loaded on top of the gradient which was then centrifuged in a Beckman SW41 rotor at 30,000 × rpm for 17–18 h at 4 °C. Twelve fractions were collected from the top and the proteins were precipitated by adding TCA to 10%. Samples were then resolved via SDS-PAGE and electro-transferred prior to immunoblotting.

### Native IPs

Cells grown in tissue culture dishes were washed with ice-cold PBS, then lysed using the following buffer: 20 mM HEPES–KOH, pH 7.2, 50 mM potassium acetate, 2 mM EDTA, 200 mM sorbitol, 0.1% Triton X-100 containing Halt™ Protease Inhibitor Cocktail. The lysates were first cleared by centrifugation at 10,000×*g* for 10 min, the resulting supernatant was transferred to a fresh tube to which protein A–Sepharose beads (Amersham Biosciences), pre-equilibrated in lysis buffer, were added to pre-clear by incubating for 30 min at 4 °C on a rotating wheel. After removal of the beads by centrifugation at 10,000×*g* for 30 s, appropriate antibodies were added and incubated for 2 h at 4 °C on a rotating wheel, and this was followed by the addition of protein A–Sepharose for antibody capture. After rotation for 1 h at 4 °C, the beads were collected by centrifugation, washed 4× with lysis buffer, desiccated in a SpeedVac and resuspended into LDS sample buffer prior to electrophoresis.

### Cell culture

All cell lines were maintained in complete medium [DMEM/high-glucose medium containing 10% foetal bovine serum (FBS), 2 mM l-glutamine, 100 units/mL penicillin, 100 mg/mL streptomycin, and 250 ng/mL Amphotericin B (all from Life Technologies)]. HeLa and SH-SY5Y cells stably expressing PLD3–GFP were grown in complete medium that additionally contained 0.4 mg/mL geneticin (Life Technologies). To eliminate possible mycoplasma contamination cells were treated with Plasmocin (Invivogen, San Diego, CA, USA).

### Transfection

Transfection of cells with constructs was carried out using jetPEI (Polyplus—transfection) following manufacturer’s instructions. Cells were harvested for further downstream applications 48 h post-transfection. Stable cell lines were established by selecting cells resistant to G418 treatment.

For siRNA transfection, cells were seeded in either 24-well plates or 6-well plates to 30–40% confluency 24 h post-seeding. At this point, ON-TARGETplus SMARTpool siRNAs (final concentration of 10 nM) were delivered to cells using the Lipofectamine™ RNAiMAX Transfection Reagent (Life Technologies), following the manufacturer’s instructions. After transfection cells were incubated in complete medium. Silencing of desired genes was carried out over 72 h.

### Immunofluorescence

Cells grown on glass coverslips were washed with PBS and then fixed in 4% (w/v) paraformaldehyde for 20 min at 37 °C. Subsequently, coverslips were rinsed 2–3 times with DMEM and left shaking gently for 15 min to remove all traces of paraformaldehyde before subsequent processing. Cells were permeabilised with 0.1% (v/v) Triton X-100 in immunofluorescence blocking buffer (IF block) (3% BSA in 1× PBS) for 15 min at room temperature, rinsed with 3 × 2 ml TBS (total wash time 20 min) and incubated for 1 h with IF block. Coverslips were then incubated with primary antibodies diluted in IF block for 1–2 h at room temperature, rinsed with 4 × 2 ml TBS, and incubated for 30 min–2 h in the dark with secondary antibodies, also diluted in IF block. Secondary antibodies were conjugated to Alexa Fluor^®^ 488, 555, 594 or 647 and obtained from Thermo Fisher. Coverslips were again rinsed with 4 × 2 ml TBS before mounting onto slides with ProLong Gold (Life Technologies). Images were captured using a Zeiss Axioimager Z2 Upright Microscope or a Zeiss LSM880 Confocal Microscope with a 63 × 1.4 N.A. oil-immersion objective lens and Immersol 518F immersion oil (all from Zeiss). Images were processed and analysed using the ZEISS Zen software, ImageJ and Volocity. For quantitation of tubulation phenotypes, 5–10 fields containing 10–25 cells were imaged using a Zeiss Axioimager Z2 microscope. Cells were counted and scored for the presence of tubules by visual inspection.

### Structured illumination microscopy (SIM)

Cells grown on 18-mm square high-performance coverslip (No. 1.5, Zeiss) for 24 h were washed with PBS before fixing in 4% formaldehyde in PBS at 37 °C for 15 min. Subsequent permeabilisation, blocking, staining and mounting steps were as for regular immunofluorescence (see above). SIM was performed on a Zeiss Elyra PS1 microscope at 23 °C using a 63 × 1.4 N.A. plan-apo objective lens (Zeiss) and Immersol 518F (Zeiss) immersion oil. Image stacks were acquired using the Zeiss ZEN Black 2012 software for five grating phases and five grating rotations at z positions spaced 110 nm apart. Channel alignment information was created using a 3D array of multispectral beads imaged with the same instrument settings. Structured illumination processing and channel alignment were performed using the ZEN Black ELYRA edition software. The presented data are a region of a single slice out of a z-stack.

### High-content imaging

Cells plated in 24-well plates were fixed and stained as for immunofluorescence. In a final staining step cells were labelled with a whole cell stain (Whole Cell Stain Blue, Thermo Fisher). Images were acquired on a CellInsight CX7 automated microscope and analysed using the HCS studio software and its spot detector bio-application. At least 400 cells (“objects” defined by the whole cell stain) were imaged per well. The whole cell stain, Alexa Fluor^®^ 488, 555, or 647 images were acquired sequentially using a single multi-pass filter set. A 20× objective lens was used.

### FACS

Cells were resuspended in complete medium containing 20% FCS (enhanced medium), and GFP-expressing cells were sorted using the BD Influx cell sorter. Cells were allowed to recover in enhanced medium for 24 h and then used for further applications.

## Results

**Fig. 1:**
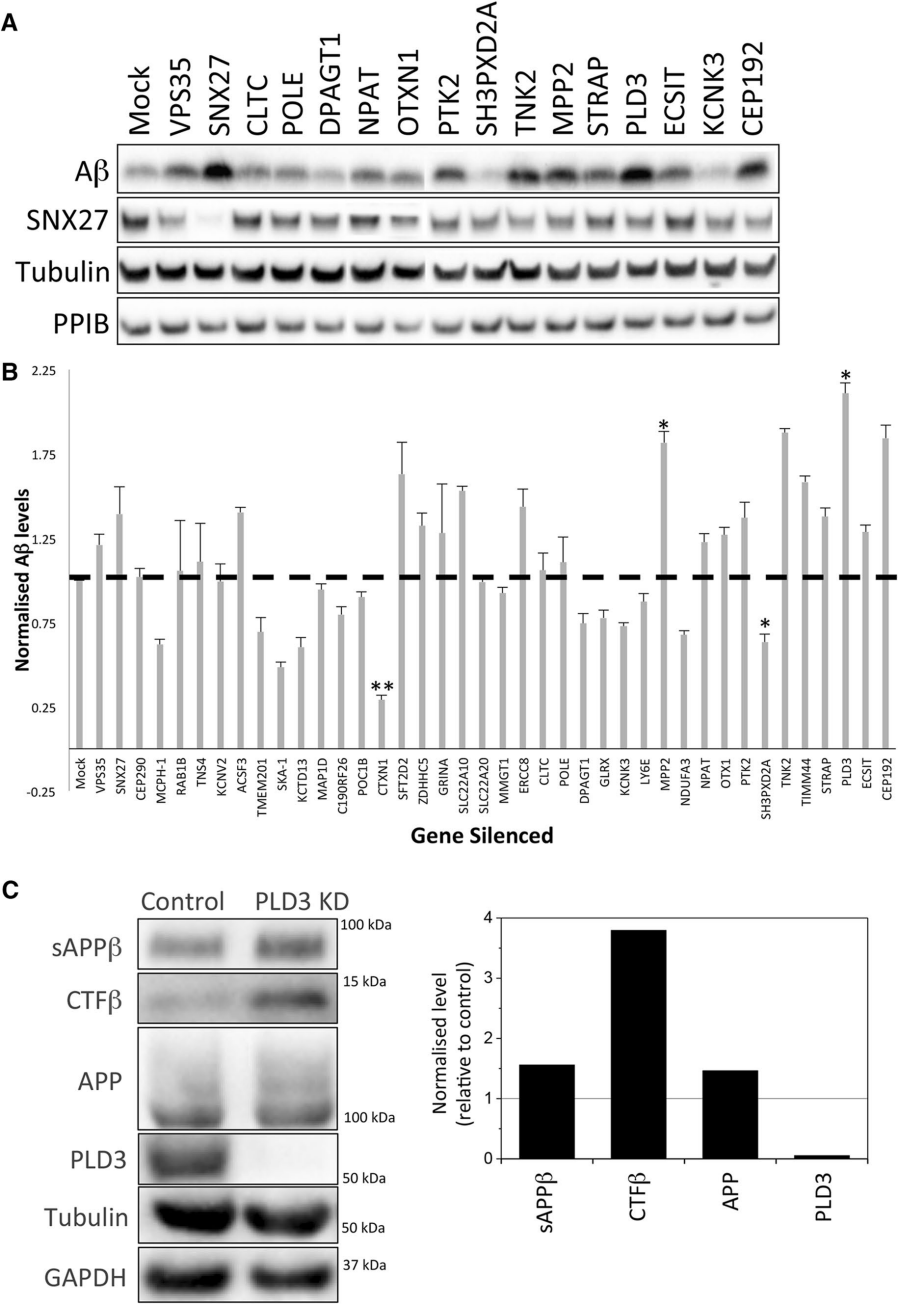
PLD3 knockdown increases secreted Aβ levels. **a** Cell culture supernatants were collected from control or siRNA-treated HEK293 cells stably expressing swAPP and analysed by Western blotting for Aβ. Corresponding cell lysates were also analysed by Western blotting for SNX27, tubulin, PPIB and GAPDH (not shown). **b** Quantitation of Aβ levels detected by Western blotting as in **a** and normalised to the three loading controls. Error bars indicate SD of three experiments. Statistical significance was determined by Student’s *t* test in Microsoft Excel (**P < 0.01, *P < 0.05). **c** Additional Western blotting of APP, Aβ and CTFβ levels in lysates from control and PLD3 knockdown cells. Tubulin and GAPDH are loading controls. The band intensities have been quantified and are shown in graphical form next to the blot

To identify genes that have a role in APP processing, we selected ~ 40 high-confidence validated hits from the genome-wide siRNA screen [21] and tested each for a role in regulating APP processing to Aβ by silencing the expression of the gene-of-interest by siRNA. As positive controls we also silenced expression of Vps35 and Snx27 individually as both have been shown to cause increased Aβ production when silenced [18, 22]. The level of the Aβ peptide secreted into the media was assessed by Western blotting. Cell lysates were also generated and intracellular proteins (e.g. Snx27) were detected by Western blotting. In Fig. 1a, data from the Aβ secretion assay are shown for 14 of the endosome-to-Golgi retrieval screen hits. Levels of Aβ vary across the different knockdowns with the PLD3 and MPP2 knockdowns generating phenotypes similar to the Snx27-positive control whilst the SH3PXD2 and KCNK3 knockdowns appear to reduce secreted Aβ. In Fig. 1b, levels of secreted Aβ from ~ 40 of the high-confidence hits are shown graphically from triplicate experiments after normalisation to loading controls. The PLD3 knockdown gives the most pronounced increase in Aβ levels and was selected for further study. Increased processing of APP to Aβ would be expected to result in increased levels of sAPPβ and the C-terminal fragment resulting from beta cleavage (Ctf-β) and this is what we observe in a PLD3 knockdown (Fig. 1c). To confirm that the increased Aβ detected after PLD3 knockdown is not the result of off-target effects we analysed each of the four siRNA comprising the PLD3-targetting SmartPool siRNA. Two of the four siRNA (sequences 10 and 11) generated increased Aβ similar to the SmartPool siRNA (see Supplemental Figure S1).

**Fig. 2:**
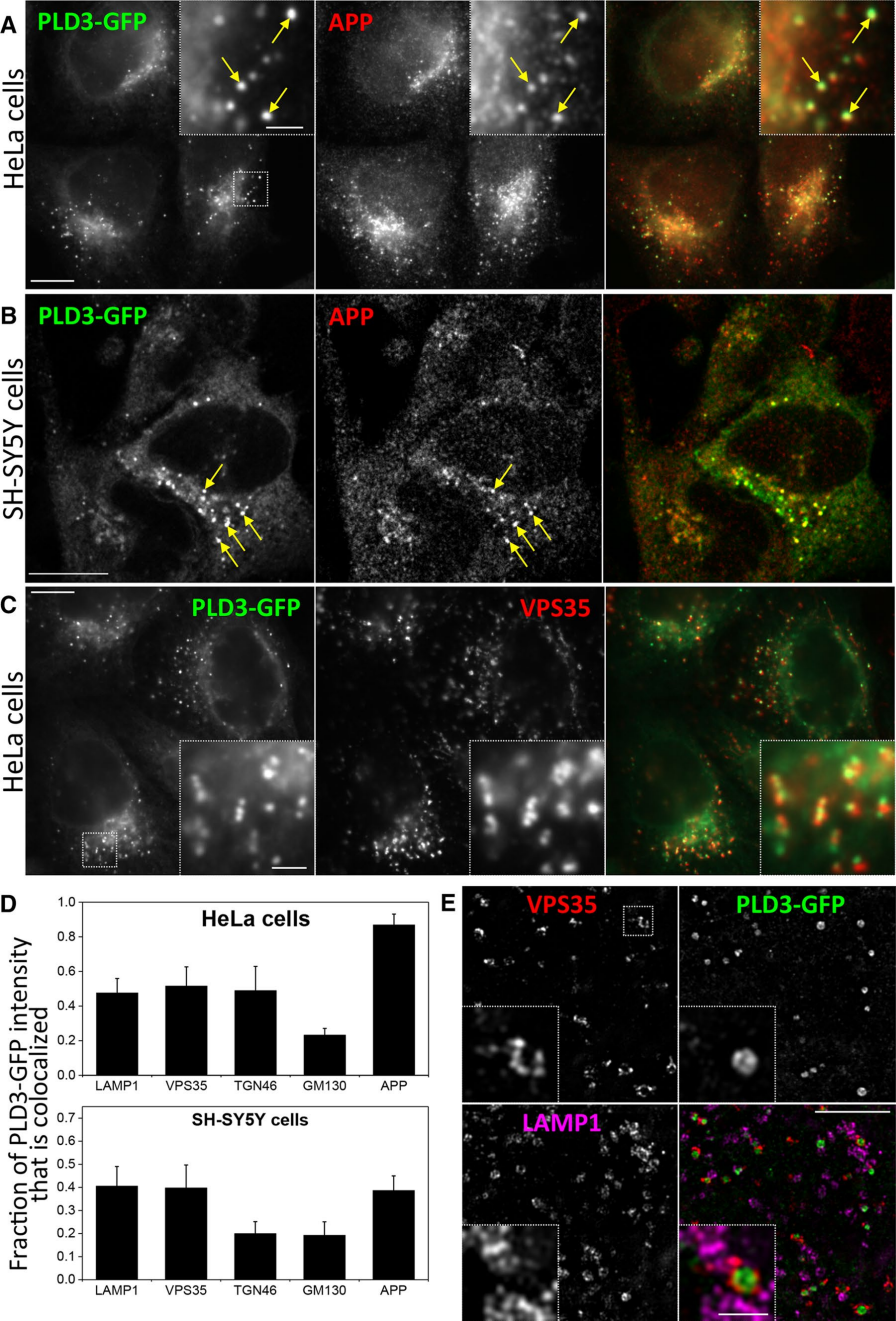
PLD3–GFP co-localises with APP in the endo-lysosomal system. HeLa cells (**a**) or SH-SY5Y (**b**) cells stably expressing PLD3–GFP were fixed and stained for GFP and APP and imaged using full-field (**a**) or confocal (**b**) microscopy. Colocalisation was observed and is highlighted in the enlarged inset areas. **c** HeLa cells stably expressing PLD3–GFP were fixed and stained for GFP and VPS35 and imaged using full-field microscopy. Colocalisation was observed and is highlighted in the enlarged inset areas. **d** The colocalisation of PLD3–GFP with markers of the Golgi and post-Golgi endo/lysosomal system was quantified using the M1 coefficient of colocalisation. **e** The localisation of PLD3–GFP was analysed in detail by structured illumination microscopy. A single 110-nm-thick section is shown. GFP signal is observed on the lumenal side of VPS35 endosomes, consistent with the predicted type II membrane topology of PLD3–GFP. Scale bars **a**–**c** 10 μm, insets 2 μm, **e** 5 μm, inset 1 μm

If PLD3 is influencing the processing of APP it would be predicted to localise to compartments that APP may traffic through, e.g. endosomes or the Golgi complex. To investigate the localisation of PLD3, a GFP tag was added to the C terminus of PLD3 and HeLa cells were transfected with the PLD3–GFP construct. PLD3 is a type II membrane protein and, therefore, the GFP moiety will be on the lumenal side of the membrane. The PLD3–GFP construct was found to colocalise with a similar construct tagged with mcherry that has been reported previously by Sleat et al. [23]. The PLD3–GFP construct also fractionated similar to endogenous PLD3 when analysed by sucrose density gradient fractionation indicating that it traffics in a manner similar to the native PLD3 protein (see Supplemental Figure S2). In Fig. 2a, HeLa cells stably expressing PLD3–GFP were fixed and labelled with antibodies against APP. We observed that a proportion of the PLD3–GFP colocalised with APP. A similar degree of colocalisation was observed for PLD3–GFP and APP in neuroblastoma SH-SY5Y cells (Fig. 2b) and PLD3–GFP was detected in endosomes positive for the retromer protein VPS35 (Fig. 2c). Quantitation of the colocalisation of the PLD3–GFP protein with endo/lysosomal- and Golgi-localised proteins including Lamp1, TGN46 and APP confirmed that PLD3–GFP was present in Golgi and post-Golgi membranes in both HeLa and SH-SY5Y cells (Fig. 2d) An examination of the PLD3–GFP localisation was conducted using super-resolution microscopy and is shown in Fig. 2e. Here the GFP fluorescence is detected inside the lumen of Vps35-positive endosomal membranes. Lamp1-positive structures are present nearby. We confirmed that PLD3 is a type II membrane protein by generating a construct where the GFP moiety is present at the N terminus and, therefore, should be orientated towards the cytoplasm and inaccessible to anti-GFP antibodies applied to the outside of unpermeabilised cells (see Supplemental Figure S3).

**Fig. 3:**
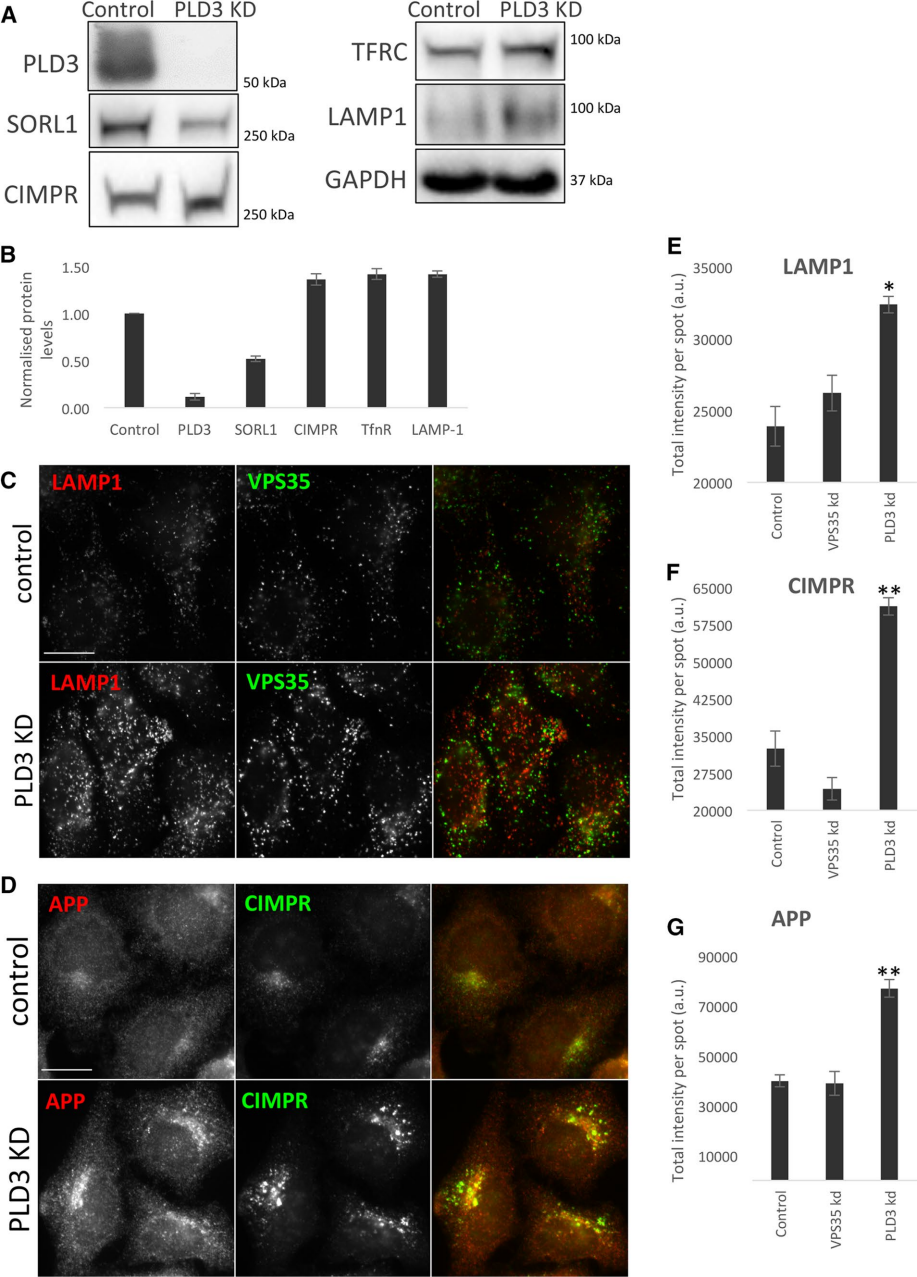
Silencing of PLD3 perturbs endosomal protein sorting. **a** Control SH-SY5Y cells and cells treated with PLD3 siRNA were lysed and analysed by Western blotting. Levels of SorL1 appear reduced whilst other proteins such as the transferrin receptor (TFRC) are modestly increased. **b** Western blot data from three independent experiments are shown graphically. **c**, **d** Control and PLD3-silenced HeLa cells were fixed and stained for various endosomal proteins, lysosomal markers and transmembrane proteins. Scale bars = 20 μm. Quantitation of the changes in immunofluorescent localization of LAMP1 (**e**), CIMPR (**f**) and APP (**g**) upon PLD3 knockdown using automated microscopy. Statistical significance was determined by Student’s *t* test in Microsoft Excel (**P < 0.01, *P < 0.05)

The colocalisation we observed between the PLD3–GFP and endosomal markers such as VPS35 indicated that PLD3 may play a role in regulating endosomal protein sorting—consistent with its identification as a novel endosome-to-Golgi retrieval gene through the genome-wide siRNA screen [21]. We, therefore, investigated the levels and localisation of several membrane proteins that transit through endosomes. In Fig. 3a, a Western blot of membrane proteins from the neuroblastoma cell line SH-SY5Y is shown. We observed that levels of SorL1 were reduced whilst other membrane proteins, e.g. the transferrin receptor (TFRC) and Lamp1 appeared increased. In Fig. 3b, Western blot data from three independent experiments have been quantified. Loss of PLD3 function by siRNA knockdown in the SH-SY5Y cells appeared to increase levels of the lysosomal marker protein, Lamp1 and this was confirmed in HeLa cells treated with siRNA targeting PLD3 expression. In Fig. 3c, control and PLD3 knockdown cells have been labelled with antibodies against Lamp1 and VPS35 or antibodies against APP and CIMPR (Fig. 3d). Using an automated microscope we quantified the fluorescence intensity after PLD3 knockdown or for comparison knockdown of the retromer protein, VPS35. We observed a gain in the Lamp1 fluorescence intensity (see Fig. 3e). A similar effect was observed for the CIMPR and APP proteins (see Fig. 3f, g). Thus, changes in membrane protein levels measured using western blot analyses of lysates from SH-SY5Y cells were recapitulated in HeLa cells using automated microscopy.

**Fig. 4:**
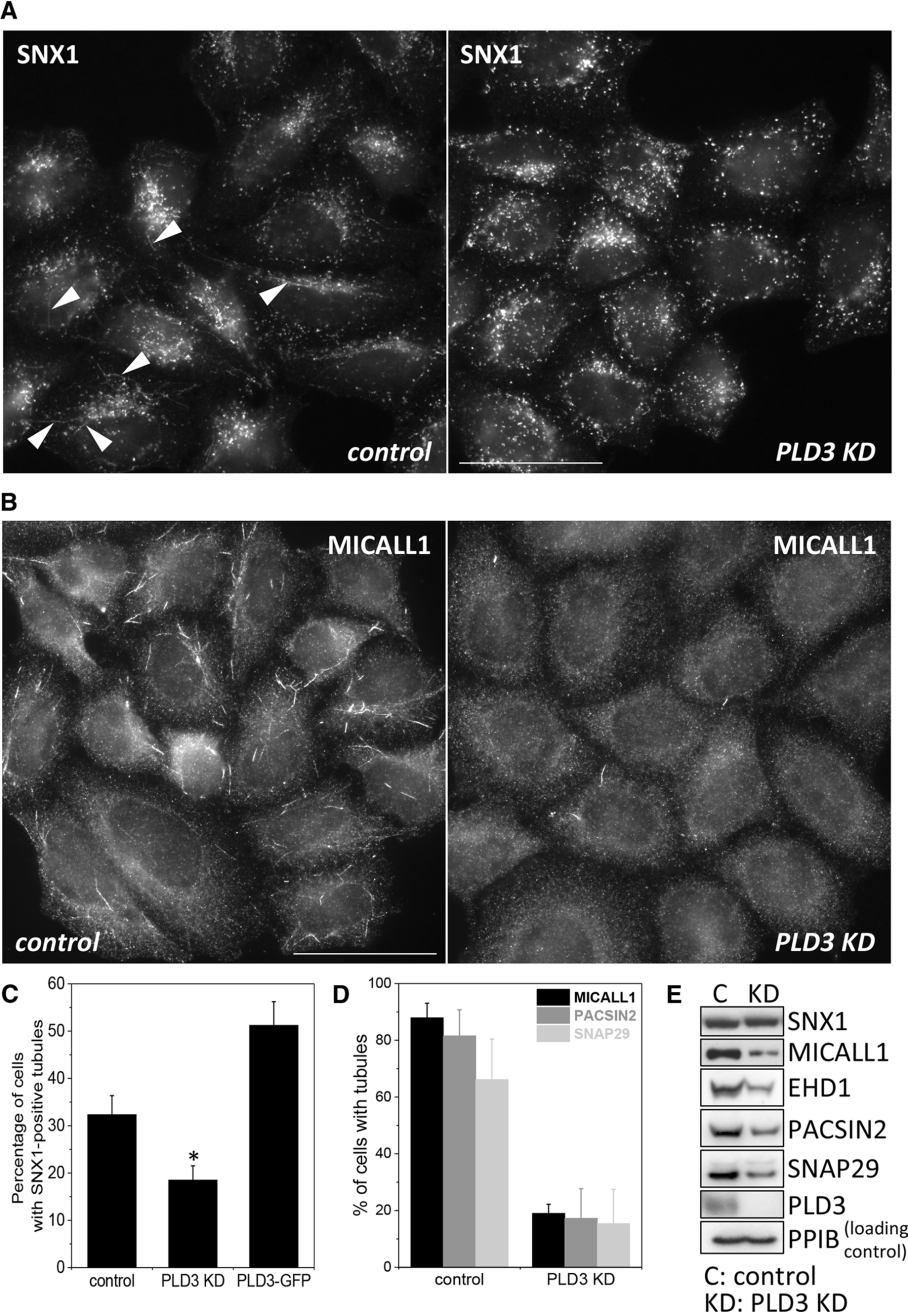
Loss of PLD3 impairs endosomal tubule formation or stability. **a** HeLa cells were treated with siRNA to abolish PLD3 expression and then fixed and labelled with anti-Snx1. Arrow heads indicate tubules that were rarely observed in PLD3 knockdown cells. Scale bar = 50 μm. **b** As in **a**, but cells were labelled with antibodies against the MICALL1 protein. Scale bar = 50 μm. **c** Snx1-decorated endosomal tubules were imaged in control HeLa cells, PLD3 siRNA-treated HeLa cells and HeLa cells stably expressing PLD3–GFP. Tubules were quantified. The results of two independent experiments are shown (average ± SD) with more than 50 cells counted in each condition in each experiment. Statistical significance was determined by Student’s *t* test in Microsoft Excel (*P < 0.05). **d** MICALL1 tubules were counted in three independent experiments, scoring more than 75 cells each time for each condition. Tubules that were PACSIN2- or SNAP29 positive were also counted in more than 100 cells and the data presented graphically. **e** Cell lysates were analysed by Western blotting. Loss of PLD3 expression does not affect SNX1 levels but does result in reduced levels of MICALL1 and the associated proteins (i.e. EHD1, PACSIN2 and SNAP29)

In previous studies we have observed that conditions that perturbed endosomal protein sorting often lead to changes in the numbers of Snx1-positive tubules. For example, knockdown of the WASH complex results in increased Snx1 tubules but loss of EHD1 expression has the opposite effect. [24, 25]. Whilst we were examining PLD3 knockdown cells for changes in membrane protein localisation, we noticed that there appeared to be fewer tubular structures positive for Snx1. In Fig. 4a, control and PLD3 knockdown of HeLa cells have been labelled with antibodies against Snx1. Tubules are evident in the control cells but generally absent in the PLD3 knockdown cells. We observed a similar loss of tubules positive for MICALL1, a protein that functions with EHD1, Pacsin2 and Snap29 in mediating traffic from recycling endosomes to the cell surface [26, 27] (see Fig. 4b and Supplemental Figure S4 for images of Pacsin2- and Snap29-positive tubules). When tubule numbers were determined, the knockdown of PLD3 caused a ~ 40% reduction in Snx1 tubules whereas Snx1-tubules increased in cells stably expressing the PLD3–GFP construct. The loss of MICALL1 tubules after PLD3 knockdown was more pronounced (Fig. 4d) but it should be noted that Western blotting of cell lysates revealed a marked loss of MICALL1 protein and a reduction in the levels of proteins associated with MICALL1 including EHD1, Pacsin2 and Snap29 (see Fig. 4e). We confirmed that the loss of MICALL1 tubules after knockdown of PLD3 was not an off-target effect by analysing MICALL1 tubules in cells where single siRNA oligos were used to silence PLD3 expression (see Supplemental Figure S5).

**Fig. 5:**
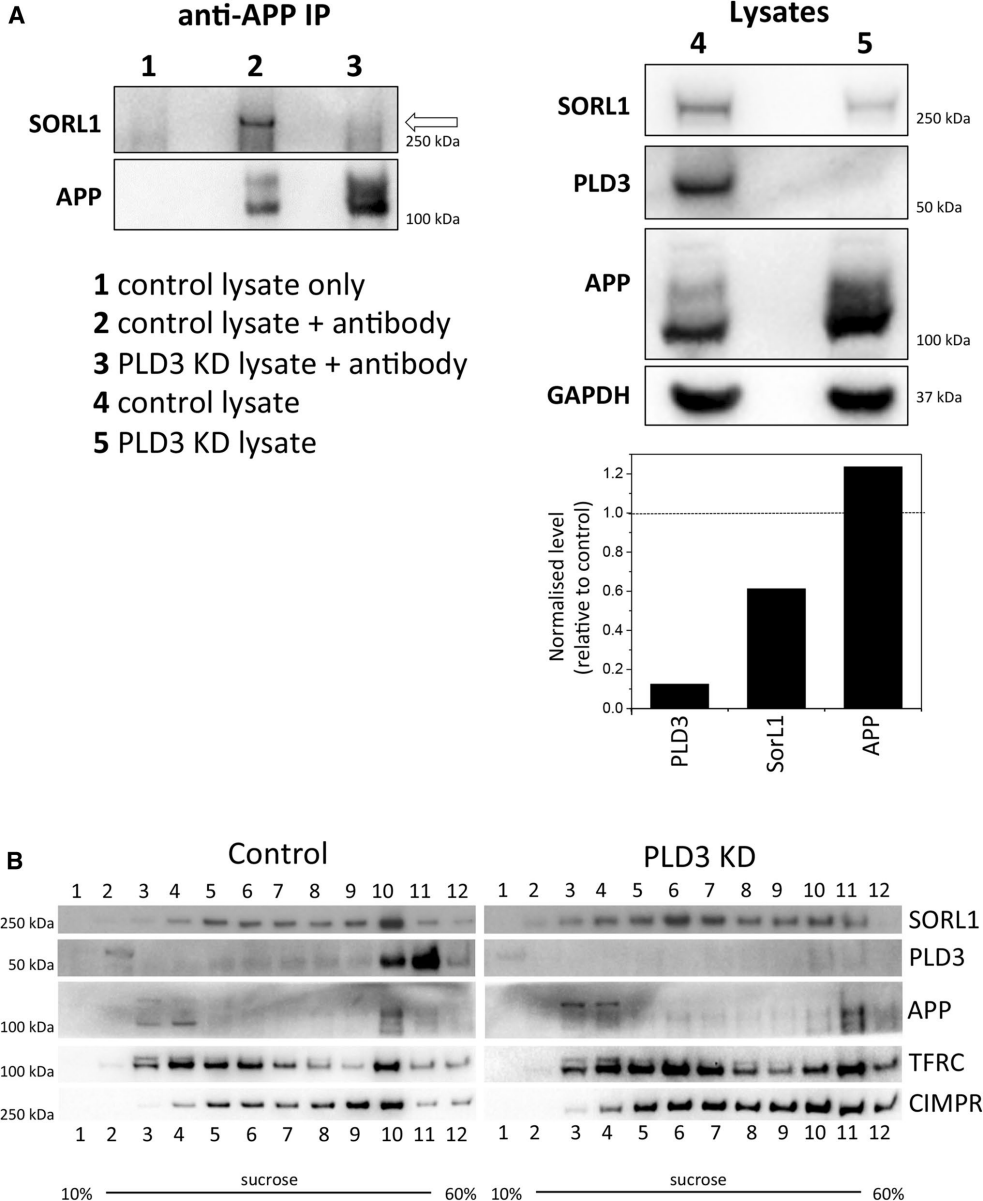
Silencing of PLD3 perturbs SorL1 association with APP and alters SorL1 subcellular distribution. **a** Control or PLD3 siRNA-treated SH-SY5Y cells were lysed and treated with monoclonal anti-APP antibody to immunoprecipitate (IP) APP. Lysates (right panel) and co-immunoprecipitated proteins (left panel) were analysed by Western blotting, the SorL1 protein is indicated by an arrow. The band intensities have been quantified and are shown in graphical form next to the blot. **b** Control and PLD3-silenced SH-SY5Y cells were lysed and subjected to centrifugation on a 10–60% sucrose gradient. Fractions (1–12) were collected and analysed by Western blotting. The fractionation profile of SorL1 is markedly altered upon PLD3 knockdown

The reduction in Snx1-positive tubules observed after loss of PLD3 expression, and the reduction in other endosomal trafficking machinery such as MICALL1 would be expected to affect many proteins that traffic through endosomes. Given that PLD3 knockdown elicited a marked increase in processing of APP to Aβ, we hypothesised that key proteins that govern APP localisation and/or processing would be similarly affected. The SorL1 protein associates with APP to regulate its localisation and processing [11–13]. We, therefore, investigated whether SorL1 could associate with APP after PLD3 knockdown. In Fig. 5a, cell lysates were treated with anti-APP antisera to recover APP and associated proteins. We observed a pronounced reduction in the amount of SorL1 associated with APP in PLD3 knockdown lysates. We next investigated the localisation of SorL1 but due to limitations of the anti-SorL1 antisera we could not determine SorL1 localisation by fluorescence microscopy. Therefore, we examined the subcellular distribution of SorL1 by sucrose density gradient fractionation in control and PLD3 knockdown lysates. In Fig. 5b, after knockdown of PLD3, the SorL1 protein is shifted on sucrose density gradients being predominately present in lighter fractions (e.g. fractions 6 and 7) whereas SorL1 is generally detected in denser fractions in lysates from control cells. The distribution of the CIMPR is also altered but the transferrin receptor (TFRC) is not observably different.

## Discussion

Here we report that PLD3, a type II transmembrane protein, is an important regulator of endosomal protein sorting and loss of PLD3 function results in increased processing of amyloid precursor protein (APP) to Aβ—possibly as a consequence of the mistrafficking of SorL1. PLD3 is a member of the phospholipase D family and, therefore, predicted to function in the conversion of phosphatidyl choline (PC) to phosphatidic acid (PA) [28] but has yet to be formally shown to possess this activity.

Our studies of endosome-to-Golgi retrieval revealed PLD3 to be required for the efficient retrieval of a CD8-CIMPR reporter protein—PLD3 is one of ~ 40 high-confidence hits from a genome-wide siRNA screen for novel endosome-to-Golgi retrieval genes [21]. As endosomal protein sorting has been intimately linked with regulating APP localisation and processing, we hypothesised that any of the novel endosome-to-Golgi retrieval genes could be important regulators of APP processing. We, therefore, tested the ~ 40 high-confidence hits for a role in controlling APP processing and found that loss of PLD3 markedly increased APP processing to Aβ, even more than the knockdown of VPS35 or SNX27, both of which have been shown to regulate APP processing [18, 22]. Interestingly, mutations in PLD3 have been linked to Alzheimer’s disease [29, 30] although this has become somewhat controversial with subsequent studies refuting the initial report [31–34]. The genome-wide screen we undertook for novel endosome-to-Golgi retrieval genes was an unbiased gene-by-gene search for new players in endosomal protein sorting and revealed a role for PLD3 [21]. The examination of the ~ 40 high-confidence hits for a role in regulating APP processing that we report here is a similar unbiased approach.

The localisation of PLD3 was determined to be endosomal and, at least partially, lysosomal. The localisation of PLD3 to lysosomes is consistent with other reports describing the localisation of PLD3 [23]. We, however, observed a significant pool of the PLD3 protein in structures that were positive for retromer proteins (i.e. Vps35), and significantly, also positive for APP. Thus, it seems likely that PLD3 traffics through the post-Golgi endocytic system and may, therefore, have wide-ranging and pleiotropic effects on endosomal protein sorting. Indeed loss of PLD3 function did result in changes in levels of several membrane proteins that traffic through endosomes including the transferrin receptor, the CIMPR and Lamp1. The levels of other proteins that operate in endosomal protein sorting also appeared changed such as MICALL1. It has been reported that the coiled-coil domain of MICALL1 binds to phosphatidic acid (PA) [27] and in doing so provides a key interaction between recycling endosomes and the MICALL1 complex that includes EHD1 and Pacsin2 (also known as Syndapin2).

Thus, the loss of PLD3 may be affecting proteins such as MICALL1 through production of PA. It should be noted, however, that the predicted catalytic domain of PLD3 is on the lumenal side of the protein and thus if PLD3 is responsible for PA production on the cytoplasmic face of endosomes, a lipid flippase may be required to translocate the PA from the lumenal side to the cytoplasmic side. There have been reports recently from studies in yeast that the Neo1 lipid flippase localises to endosomes and is trafficked by Snx3, a retromer-associated protein [35]. Thus, it is plausible that PLD3 exerts it effects on endosomal protein sorting through its function as a phospholipase D enzyme. It does not, however, appear to be essential for the production of lysobisphosphatidic acid (LBPA). The LBPA lipid has been reported to be a marker of late endosomes and lysosomes and has been linked with multivesicular body formation [36] but we did not detect any significant changes in LBPA levels in cells treated with siRNA to knockdown PLD3 (see Supplemental Figure S6).

Loss of PLD3 function did induce a reduction in Snx1-positive tubules, altered SorL1 and CIMPR distribution on sucrose density gradients and, most tellingly, resulted in a profound reduction in the amount of SorL1 associated with APP. These observations can explain why loss of PLD3 results in increased processing of APP to Aβ. The reduced Snx1 tubules would be predicted to impair the endosome-to-Golgi trafficking of both SorL1 and CIMPR resulting in their mislocalisation. Thus, APP trafficking would be impacted and increased processing to Aβ the end result. Other effects of the loss of PLD3 (e.g. reduced MICALL1) could compound the endosomal protein sorting defect and also lead to altered APP processing. It should, however, be noted that genetic knockout of PLD3 in mice did not result in altered APP processing or increased Aβ levels [34]. Why the knockout of PLD3 behaves differently from the knockdown is not clear but it is possible that some form of adaptation to the loss of PLD3 has occurred in the knockout mice, possibly through upregulation of phospholipase D family members, PLD1 or PLD2—adaptation that may not occur in the time frame of an RNAi-mediated knockdown.

## Electronic supplementary material

Below is the link to the electronic supplementary material.

Supplementary material 1 (PDF 1405 kb)

## Abbreviations

AD: Alzheimer disease
APP: Amyloid precursor protein
CIMPR: Cation-independent mannose 6-phosphate receptor
GFP: Green fluorescent protein,
LOAD: Late-onset Alzheimer disease
PLD: Phospholipase D
Snx: Sorting nexin
SorL1: Sortilin-like 1
TGN: Trans-Golgi network
Vps: Vacuolar protein sorting
