# Supplementary material for: Analysis of novel endosome-to-Golgi retrieval genes reveals a role for PLD3 in regulating endosomal protein sorting and amyloid precursor protein processing
Source: Cell Mol Life Sci. 2018 Jan 24;75(14):2613–25. doi: 10.1007/s00018-018-2752-9 (PMC6003983; doi:10.1007/s00018-018-2752-9)
Supplement: Supplementary file 1 — Supplementary material 1 (PDF 1405 kb) [file 18_2018_2752_MOESM1_ESM.pdf]

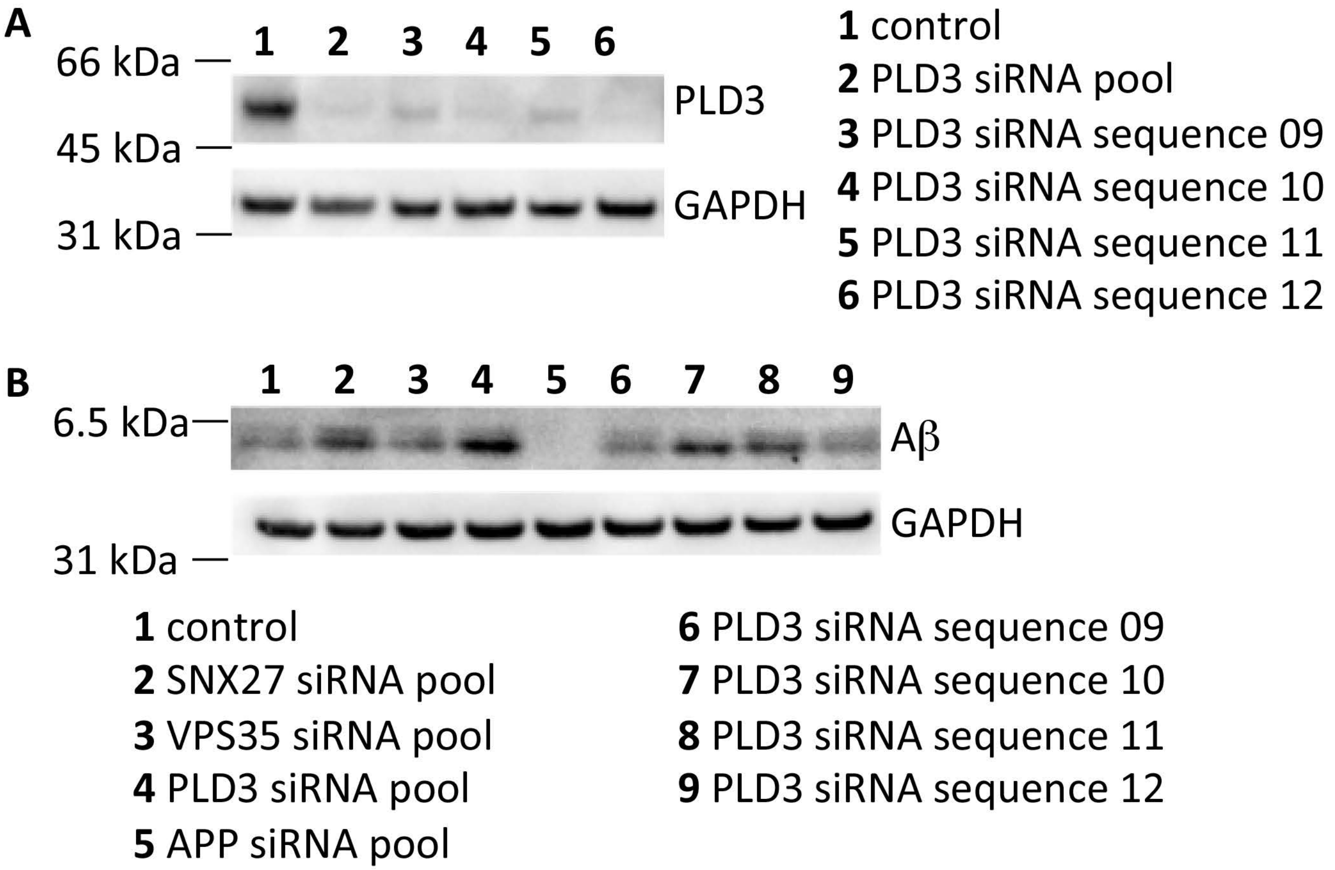
A
1 2 3 4 5 6
66 kDa
45 kDa
31 kDa
PLD3
GAPDH
1 control
2 PLD3 siRNA pool
3 PLD3 siRNA sequence 09
4 PLD3 siRNA sequence 10
5 PLD3 siRNA sequence 11
6 PLD3 siRNA sequence 12
B
1 2 3 4 5 6 7 8 9
6.5 kDa
31 kDa
Aβ
GAPDH
1 control
2 SNX27 siRNA pool
3 VPS35 siRNA pool
4 PLD3 siRNA pool
5 APP siRNA pool
6 PLD3 siRNA sequence 09
7 PLD3 siRNA sequence 10
8 PLD3 siRNA sequence 11
9 PLD3 siRNA sequence 12

Figure S1

A

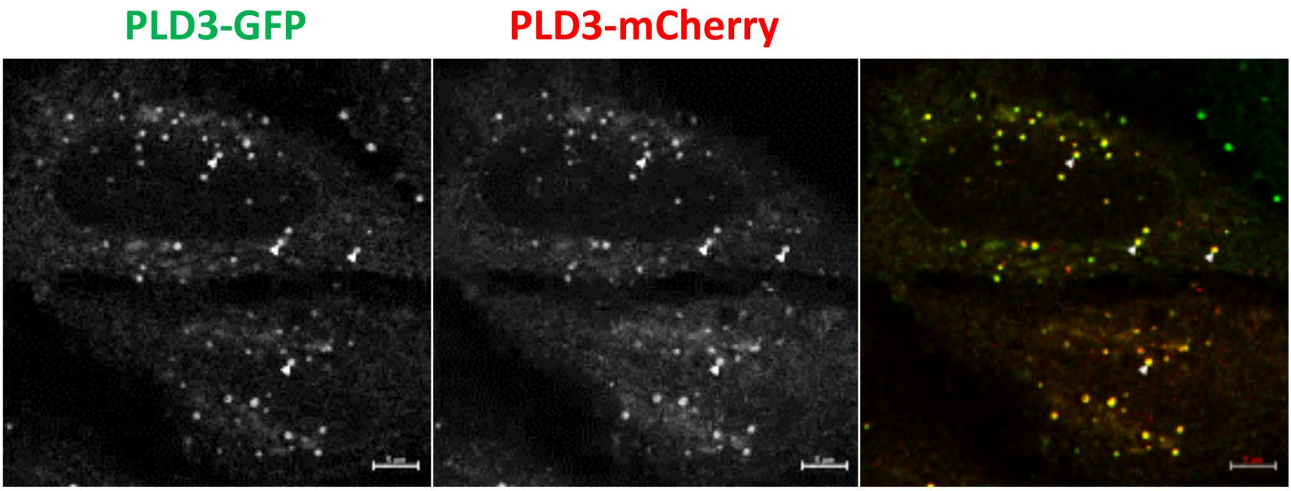
PLD3-GFP
PLD3-mCherry

B

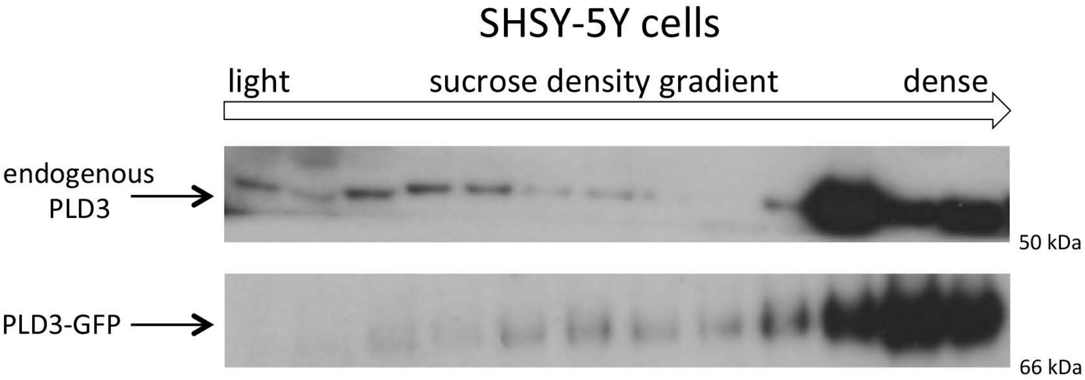
SHSY-5Y cells
light
sucrose density gradient
dense
endogenous PLD3
PLD3-GFP
50 kDa
66 kDa

Figure S2

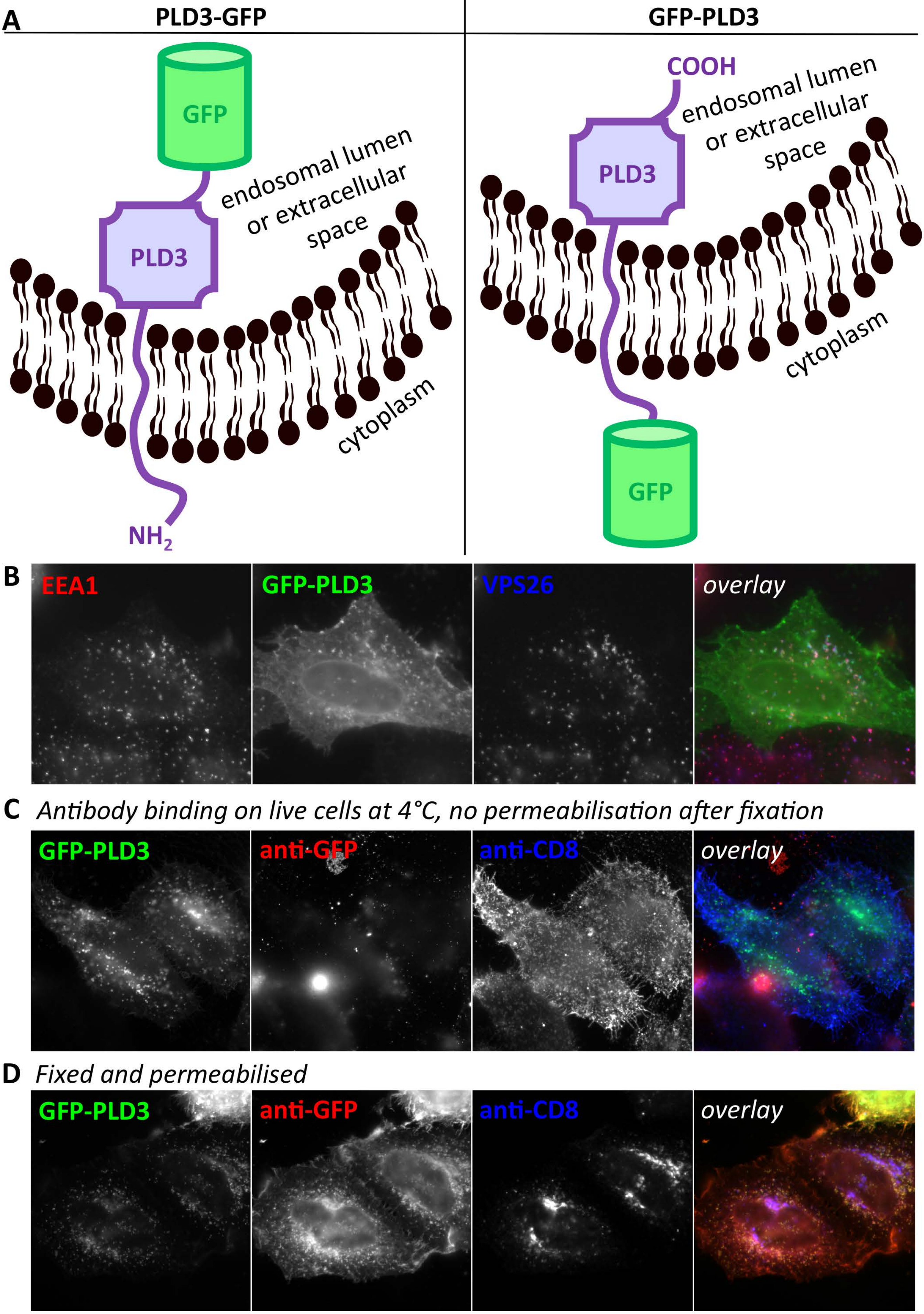
A
PLD3-GFP
GFP
PLD3
endosomal lumen
or extracellular
space
cytoplasm
NH2
GFP-PLD3
COOH
PLD3
endosomal lumen
or extracellular
space
cytoplasm
GFP
B
EEA1
GFP-PLD3
VPS26
overlay
C
Antibody binding on live cells at 4°C, no permeabilisation after fixation
GFP-PLD3
anti-GFP
anti-CD8
overlay
D
Fixed and permeabilised
GFP-PLD3
anti-GFP
anti-CD8
overlay

Figure S3

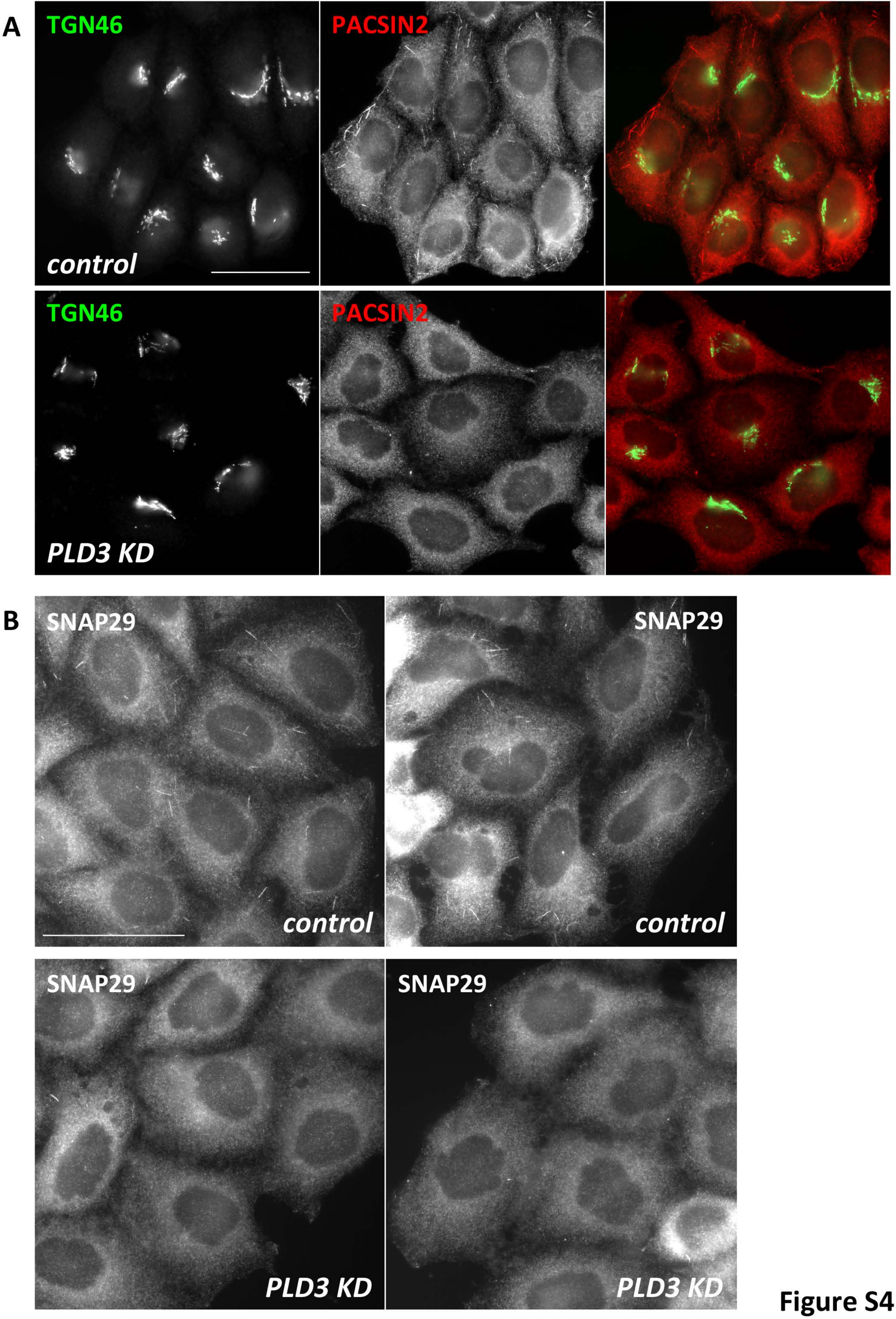
A
TGN46
PACSIN2
control
TGN46
PACSIN2
PLD3 KD
B
SNAP29
SNAP29
control
control
SNAP29
SNAP29
PLD3 KD
PLD3 KD

Figure S4

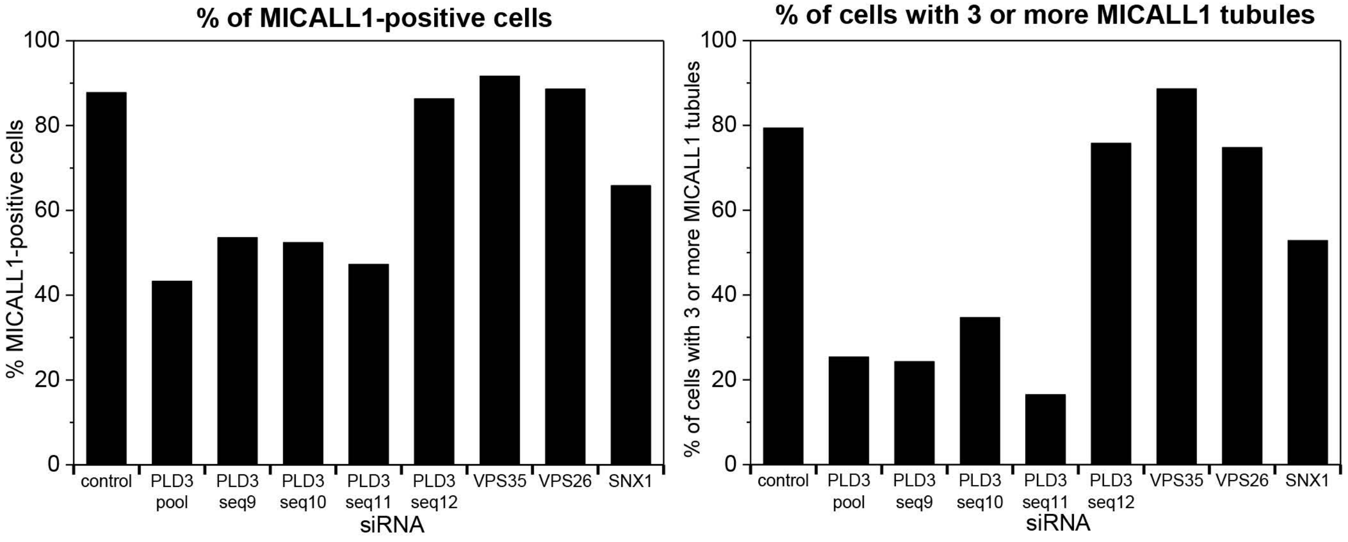
% of MICALL1-positive cells
% MICALL1-positive cells
100
80
60
40
20
0
control
PLD3 pool
PLD3 seq9
PLD3 seq10
PLD3 seq11
PLD3 seq12
VPS35
VPS26
SNX1
siRNA
% of cells with 3 or more MICALL1 tubules
% of cells with 3 or more MICALL1 tubules
100
80
60
40
20
0
control
PLD3 pool
PLD3 seq9
PLD3 seq10
PLD3 seq11
PLD3 seq12
VPS35
VPS26
SNX1
siRNA

Figure S5

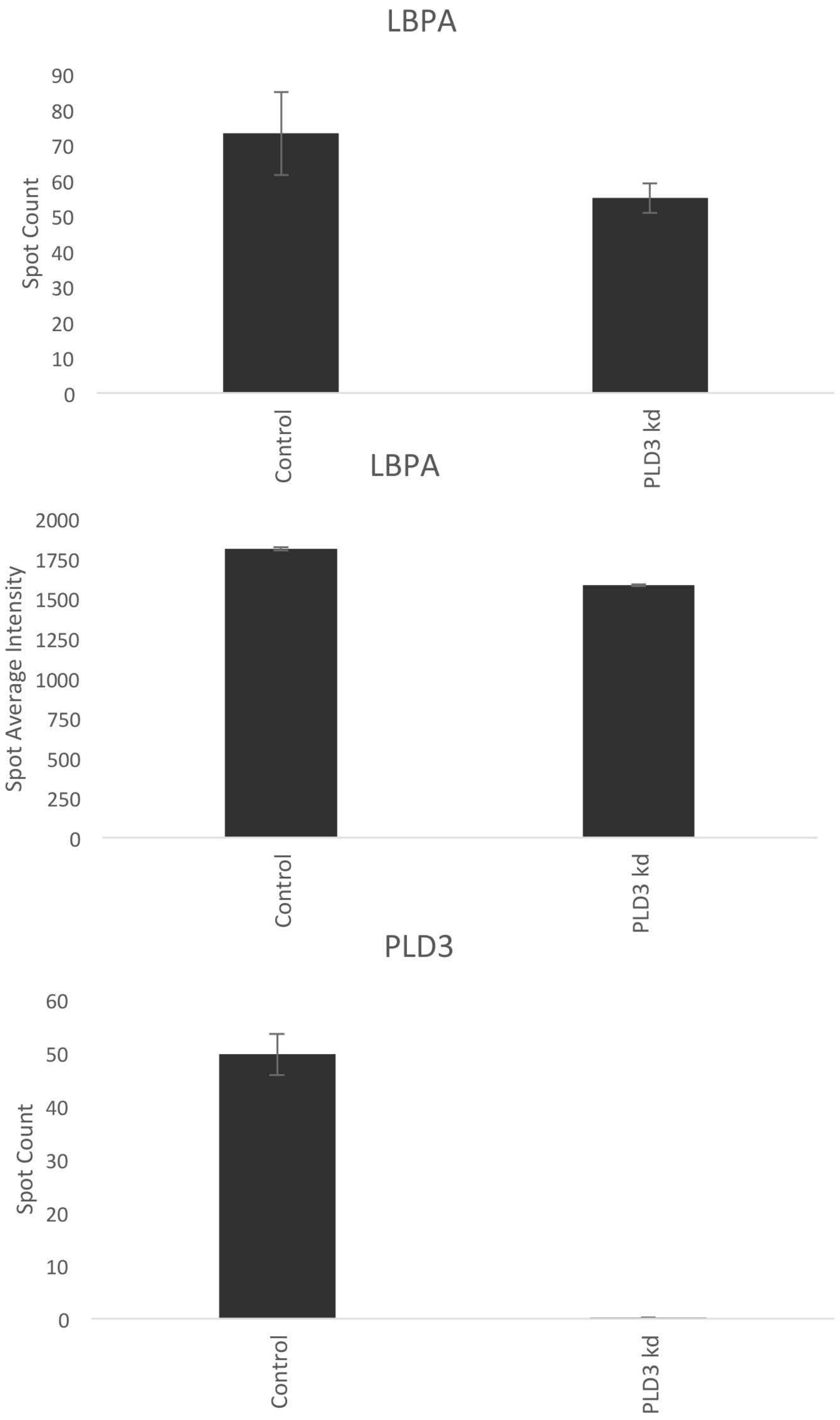
LBPA
Spot Count
0
10
20
30
40
50
60
70
80
90
Control
PLD3 kd
LBPA
Spot Average Intensity
0
250
500
750
1000
1250
1500
1750
2000
Control
PLD3 kd
PLD3
Spot Count
0
10
20
30
40
50
60
Control
PLD3 kd

Figure S6

Supplemental Figure S1. (A) SH-SY5Y cells were treated for 72 hours with siRNA against PLD3, either a pool of 4 sequences or a single sequence. Lysates were generated and probed by Western blotting for PLD3 and GAPDH (loading control). (B) HEK293 cells stably expressing swAPP were mock treated (control) or treated with siRNAs as indicated, and secreted Aβ was detected by Western blotting. Two of the 4 sequences in the PLD3 siRNA pool show marked increase in Aβ secretion when compared to control.

Supplemental Figure S2. (A) HeLa cells stably expressing PLD3-GFP were transfected with PLD3-mCherry and observed by live cell confocal microscopy. A single frame is shown, indicating the nearly complete overlap between the GFP and mCherry signals. Scale bar = 5 μm. **(B).** SHSY-5Y cells, either untransfected or stably expressing PLD3-GFP were lysed and subjected to sucrose density gradient fractionation. Fractions have been analysed by western blotting using anti-PLD3. The PLD3-GFP protein behaves similarly to the endogenous PLD3 protein.

Supplemental Figure S3. Topology of GFP-PLD3 and PLD3-GFP. **(A)** Cartoon depicting the predicted topology of the GFP-PLD3 and PLD3-GFP fusion constructs. **(B)** HeLa cells transiently transfected with GFP-PLD3 were fixed and labelled with antibodies against EEA1 and retromer subunit VPS26. Broadly similar to PLD3-GFP (See Figure 2) GFP-PLD3 localizes to EEA1- and VPS26-positive endosomes; in addition plasma membrane localisation is seen. **(C)** HeLa cells stably expressing CD8-CIMPR were transiently transfected with GFP-PLD3. After 24 hours cells were incubated for 30 minutes with anti-CD8 and anti-GFP antibodies at 4°C before fixing and incubation with fluorescently labelled secondary antibodies. While the anti-CD8 antibody binds to the extracellular CD8 epitope, anti-GFP is unable to bind to its epitope, indicating it is present in the cytosol. **(D)** In contrast, when similar cells (stably expressing CD8-CIMPR and transiently expressing GFP-PLD3) are fixed and permeabilised before labelling with anti-CD8 and anti-GFP antibodies, both antibodies bind to their epitopes.

Supplemental Figure S4. Loss of PLD3 results in reduced PACSIN2 and SNAP29 tubules. Control and PLD3 KD HeLa cells were labelled with antibodies against PACSIN2 and TGN46 (**A**) or SNAP29 (**B**). Almost no tubular structures positive for PACSIN2 or SNAP29 were visible after PLD3 KD. Scale bars = 50 μm.

Supplemental Figure S5. HeLa cells were mock treated (control) or treated for 48 hours with the indicated siRNAs, fixed, antibody-labelled and imaged. Per condition more than 90 cells were scored for the presence of MICALL1-positive tubules. Knockdown of PLD3 using either the pool of siRNA sequences or 3 of the 4 single siRNA sequences reduces the percentage of cells with MICALL1-positive tubules (left graph) and especially the percentage of cells with 3 or more MICALL1-positive tubules (right graph).

Supplemental Figure S6. Loss of PLD3 does not affect levels of lysobisphosphatidic acid (LBPA). HeLa cells stably expressing PLD3-GFP were treated with siRNA to silence PLD3 expression. After fixation, the cells were labelled with monoclonal anti-LBPA and then imaged using an automated microscope. No significant changes in spot number (**A**) or average intensity (**B**) was observed for LBPA after loss of PLD3 expression whereas the number of PLD3 spots was reduced to virtually nil (**C**).
